# Trends in self-rated health among the elderly population in Germany from 1995 to 2015 – the influence of temporal change in leisure time physical activity

**DOI:** 10.1186/s12889-020-8218-7

**Published:** 2020-01-28

**Authors:** Stefanie Sperlich, Johannes Beller, Jelena Epping, Juliane Tetzlaff, Siegfried Geyer

**Affiliations:** Hannover Medical School, Medical Sociology Unit, Carl-Neuberg-Str. 1, 30625 Hannover, Germany

**Keywords:** Time trend, SRH, Leisure time physical activity, Mediation

## Abstract

**Background:**

Against the backdrop of rising statutory retirement age in Germany, we analyzed time trends in self-rated health (SRH) among the elderly population between 50 and 70 years of age and explored the mediating role of leisure time physical activity (LTPA) on the relationship between time period and self-rated health (SRH).

**Methods:**

We used longitudinal survey data (*n* = 23,161) from a national panel study (GSOEP) to analyze time trends in SRH and regular LTPA (at least once a week) by means of Generalized Estimation Equation (GEE) analysis for logistic regression. The Karlson-Holm-Breen (KHB) method was applied for decomposing trend effects into direct and indirect parts via LTPA. In addition to odds ratios (OR), we illustrated the results by means of predicted probabilities and average partial effects (APE).

**Results:**

Over time, the predicted probabilities of good SRH and regular LTPA increased while those of poor SRH decreased. After adjusting for socioeconomic status (SES) 53.4% of the trend in good SRH in women (OR = 1.34 / APE = 6.8%-points) could be attributed to the rise in regular LTPA. In men, the remaining smaller effect (OR = 1.13 / APE = 2.7%) could be fully assigned to temporal changes in regular LTPA. With respect to poor health we found a suppression effect of LTPA in the adjusted model, indicating that without improvements in regular LTPA over time an increase in poor SRH would have occurred.

**Conclusions:**

The increase of regular LTPA accounted for improved SRH from 1995 to 2015 among the elderly, indicating that promoting LTPA might be a key factor to raise healthy working life expectancy.

## Background

The compression of morbidity hypothesis originally proposed by Fries [1] stated that better health care, an active lifestyle, and advances in preventive health behavior would lead to increased active life expectancy and decreasing duration of morbidity and disability in the population. In support of this assumption several studies revealed a significant reduction in proportions of functional impairment and also increases of disability-free life expectancy and expected lifetime in good self-rated health (SRH) [2–8]. Pointing into the same direction, German studies reported improved SRH over time particularly in the elder population [9–13].

One major predictor of health and wellbeing is physical activity (PA). PA is a broad term that encompasses both leisure time physical activity (LTPA) as well as physical activities associated with daily life in general. While LTPA refers to recreational exercise or sport, physical activities in daily life also include activities related to regular work, housework, or transport activities [14]. Leisure time and occupational physical activity appear to have distinct effects on health: high doses of LTPA are associated with reduced risk whereas high doses of occupational physical activity are accompanied by increased risk of lifestyle-related health complaints such as cardiovascular diseases, suggesting a physical activity paradox [15].

In this study, we focused on LTPA that in previous studies is often generally defined as PA. Recent studies indicated that LTPA reduced the risk of chronic diseases [16], increased healthy and disease-free life expectancy [17] and improved cognitive function in older adults [18]. Moreover, maintaining or increasing moderate physical activity was associated with a smaller age-related decline in SRH [19], and some studies demonstrated a dose-response relationship between physical activity level and health [20, 21].

At population level LTPA tended to increase over the last decades while occupational-related physical activity seemed to be decreasing [22]. Gains in regular LTPA were reported for several countries like Finland [23], Spain [14], Sweden [24] and Denmark [25]. Hence, changes in LTPA at population level might have contributed to compression of morbidity in the recent decades. However, the majority of previous studies have focused on long-term health benefits of LTPA at individual level, for example by comparing changing rates of disability between physically active and inactive persons over time [16]. The question of whether rises in regular LTPA accounted for positive health trends at population level has rarely been investigated. One of the few studies assessing long-term trends in multimorbidity and their association with physical activity (PA) did not consider whether PA had changed over time and thus could not produce evidence for mediating effects of exercise on health trends [26]. To the best of our knowledge, the current study is the first one to investigate whether changes in SRH over time at population level can be attributed to possible changes in LTPA over time.

Previous studies suggested that the temporal developments of SRH differ across ages [9, 13, 27], thus calling for an age-specific approach in analyzing health trends. Within the context of an ageing population, the German government decided to rise the statutory retirement age gradually up to the age of 67 in order to keep the pension system financially sustainable [28]. An essential precondition for this implementation is sufficiently good health and well-being of the elder working population. However, while there is some evidence of increasing healthy life expectancy [2, 3], little is known on the possible rise of healthy working life expectancy, meaning the time spent in both work and good health [29]. Thus, we focused on individuals aged 50–70 years who are most directly affected by the pension policy. In detail, the study was guided by the following research questions:

1. Has SRH improved between 1995 and 2015 among the elderly population and has regular LTPA increased accordingly?
2. How strong is the association between regular LTPA and levels of SRH?
3. Is the time trend in SRH mediated by changes in regular LTPA?

## Methods

The data of this study were drawn from the German Socio-Economic Panel Study (GSOEP V.31), carried out by the German Institute for Economic Research. The GSOEP is a national annual survey of individuals living in private households conducted annually from 1984 onwards. SOEP uses random probability samples that draw on a nation-wide two-stage stratified sampling procedure. First, nation-wide sample points are sampled by federal state and municipality size with regional sample points ranging in size from 125 to 985 per sample. Second, within each sample point, households are sampled in a random walk procedure. Random sampling with known selection probabilities allows constructing design weights and subsample-specific cross-sectional weights. The GSOEP population is updated regularly with new survey samples to reflect changes in the German population and in order to compensate for dropouts occurring over time. Our analyses are based on a pooled dataset including the waves from 1995 to 2015, allowing for trend analysis on population level by means of cross-sectional comparisons. For displaying the sample characteristics, we used the cross-sectional weights that are assumed to produce a nationally representative sample. However, since sampling weights must be constant within subjects, we did not use them for analyzing time trends because subjects’ sampling weights were changing over time.

SOEP uses different modes of data collection with face-to-face interviewing as the default that on average takes about 50 min per household. The central survey instrument for this study is an individual questionnaire, which each adult household member is supposed to answer. A stable set of core questions is asked every year, enhanced by rotating modules on topics such as leisure time activities. Further information on GSOEP can be derived from Goebel et al. [30].

We included participants between 50 and 70 years of age. Overall, 23,161 respondents (11,553 men / 11,608 women) were observed 91,741 times (45,247 men / 46,494 women) (unweighted sample), corresponding to an average participation in four waves for both women and men (min = 1 / max = 14). The weighted sample characteristics, separated by gender and time period, are presented in Table 1. The proportion of missing values on the variables included varied between 0 and 2.2% (Table 1). Respondents with missing information were excluded.

**Table 1:** Weighted sample characteristics

	men *n* = 50,848		women *n* = 52,538	
Variable	n	%	n	%
Time points (years)				
1 (95/96/97)	10,553	20.8	10,933	20.8
2 (98/99)	7190	14.1	7309	13.9
3 (01/03)	7327	14.4	7556	14.4
4 (05/07/08)	10,994	21.6	11,440	21.8
5 (09/11)	7202	14.2	7502	14.3
6 (13/15)	7582	14.9	7798	14.8
total	50,848	100	52,538	100
missing	–	0.0	–	0.0
Equivalence income				
< 60% median	5615	11.0	7319	13.9
60% - < 150% median	33,240	65.4	34,867	66.4
≥ 150% median	11,983	23.6	10,310	19.6
total	50,838	100	52,496	100
missing	10	< 0.0	42	< 0.0
School education				
primary / no education	25,445	51.0	28,030	54.5
secondary	10,394	20.8	13,141	25.6
tertiary	9634	19.3	6503	12.7
other qualification	4417	8.9	3725	7.2
total	49,890	100	51,399	100
missing	958	1.9	1139	2.2
Nationality				
German	46,190	90.8	48,792	92.9
others	4658	9.2	3746	7.1
total	50,848	100	52,538	100
missing	–	0.0	–	0.0
Marital status				
single	9945	19.6	15,835	30.1
with partner	40,901	80.4	36,701	69.9
total	50,846	100	52,535	100
missing	2	< 0.0	3	< 0.0
Employment status				
Full time	24,491	48.2	10,329	19.7
Part time	2282	4.5	10,418	19.8
Not employed	24,075	47.3	31,790	60.5
total	50,846	100	52,538	100
missing	–	0.0	–	0.0
Physical activity				
(almost) never	24,909	49.6	25,834	50.0
serveral times a year	8836	17.6	7044	13.6
at least once a month	2894	5.8	2273	4.4
at least once a week	13,577	27.0	16,522	32.0
total	50,216	100	51,674	100
missing	632	1.2	864	1.6

*n* = number of observations

### Measures

Regular LTPA was assessed by a single item that belongs to an item set measuring different leisure time activities. Participants were asked to indicate to what extent any kind of leisure time sports activities is currently performed. Between 1995 and 2015 this question was asked 14 times in irregular intervals (from 1995 to 1999 annually, since 2001 every 2 years and additionally in 2008). The response format alternated over time between four and five categories. In order to compare the different versions, a new variable was created with the following categories: ‘(almost) never’, ‘several times a year’, ‘at least once a month’ and ‘at least once a week’. In order to stratify participants into being regular active or not we created a dichotomous variable with the coding ‘at least once a week’ versus ‘to a lesser extent’.

We used the general self-rated health status (SRH) as our health indicator for which research has demonstrated predictive validity with respect to morbidity and mortality [31, 32]. SRH was measured by asking participants to assess their health with the following question: “In general, how would you rate your current health status?”. The five original response categories (‘very good’, ‘good’, ‘satisfactory’, ‘poor’ and ‘bad’) were transformed into two binary variables indicating ‘good’ (‘very good’ / ‘good’ health versus the other categories) and ‘poor’ health (‘poor’ / ‘bad’ health versus the other categories).

Changes in SRH were analyzed with a categorical time- and a continuous trend-variable. For computing the categorical time variable panel waves between 1995 and 2015 were classified into six consecutive time periods by taking the irregular intervals in assessing LTPA into account: (1) 1995/96/97, (2) 1998/99, (3) 2001/03, (4) 2005/07/08, (5) 2009/11 and (6) 2013/15. The continuous trend variable was coded 0 for 1995 and 1 for 2015, with the years in between getting fractional values, for example 0.05 for 1996, 0.10 for 1997 and so forth. We used the categorical time variable for depicting trends in SRH and LTPA and the continuous trend variable for mediation analysis.

In mediation analysis, we controlled for income, education and occupational position as possible confounders. Each socioeconomic indicator was categorized into low, middle and high social status while occupation also includes the category ‘not employed / in retirement’.

### Statistical analyses

The analyses were performed in two steps: First, we examined the preconditions for a mediating effect of regular LTPA on time trends in SRH. At the second step it was analyzed how much of the trend in SRH is explained by regular LTPA. According to Baron and Kenny [33] three preconditions needed to be met for taking a mediator effect of regular LTPA on the temporal development of SRH into consideration: (1) a significant increase in SRH over time, (2) a significant rise of regular LTPA over time and (3) a significant effect of regular LTPA on SRH. These conditions were tested by calculating population-averaged effects using generalized equation estimating (GEE) for logistic regression. We used GEE since our aim was to analyze temporal change in the population and not to model intra-individual change that would be more accurately estimated by random-effect models [34]. GEE is an extension of standard regression analysis for panel data that allows to control for possible dependencies of observations by using a working correlation matrix [35]. We found the best fitting model according to smallest likelihood information criterion (qIC) for the independent correlation structure, indicating that adjustment for autocorrelation was not required. We tested the first precondition of mediation by regressing SRH on the time trend variable (reference = baseline 1995). The second and third premises were examined by regressing LTPA on the time trend variable and SRH on LTPA, respectively. We illustrated the results by means of predicted probabilities (margins at means) adjusted for age.

**Fig. 1:**
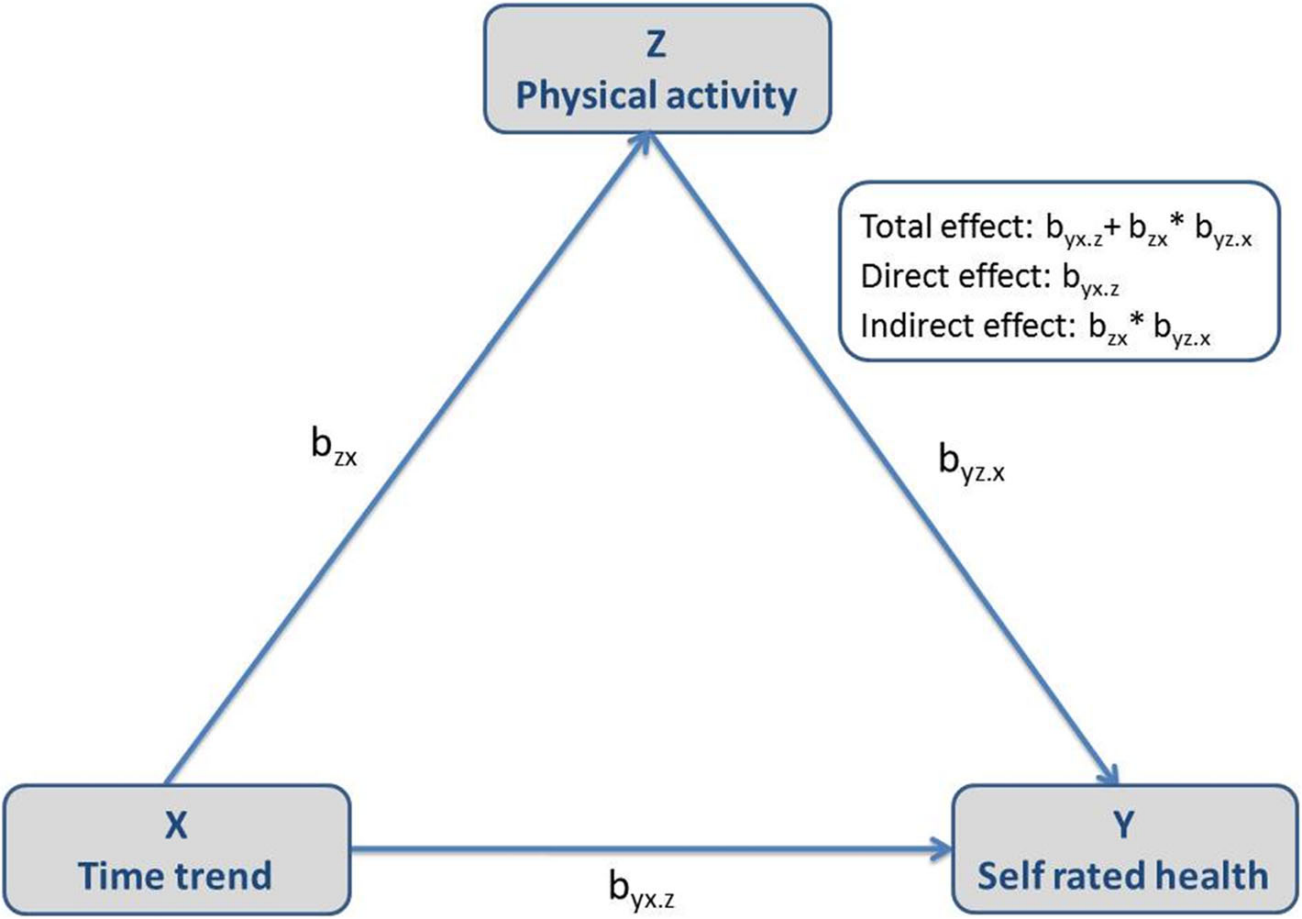
Path decomposition of total effect of time trend (X) on self-rated health (SRH) (Y) into direct and indirect effects via leisure time physical activity (LTPA) (Z)

Based on GEE logistic regression analysis, the Karlson-Holm-Breen-method (KHB-method) [36] was applied to examine how much of the total time effect on SRH is mediated by changes in LTPA over time. The KHB method extends the decomposition properties of linear models to logistic regression models by decomposing the total effect of time on SRH into a direct and indirect effect. This method ensures that the crude and adjusted coefficients presented are measured on the same scale and thus, are unaffected by the rescaling bias that arise in cross-model comparisons of non-linear models. In our case, the indirect effect is the part of the effect of time on SRH that is explained by increases in regular LTPA. The direct effect of time corresponds to the effect that is left after controlling for regular LTPA (Fig. 1). Because we suspected the decomposition to be affected by potentially confounding variables, we estimated two models: in model 1 we only adjusted for age and in model 2 we additionally controlled for income, school education and occupational position. In addition to odds ratios (OR) we reported average partial effects (APE) giving the decomposition a more substantial interpretation. APE are measured on the probability scale and estimate the average marginal effect of LTPA in the population [36]. All analyses were performed with STATA v13.1.

## Results

### Analyzing the preconditions for mediation

**Fig. 2:**
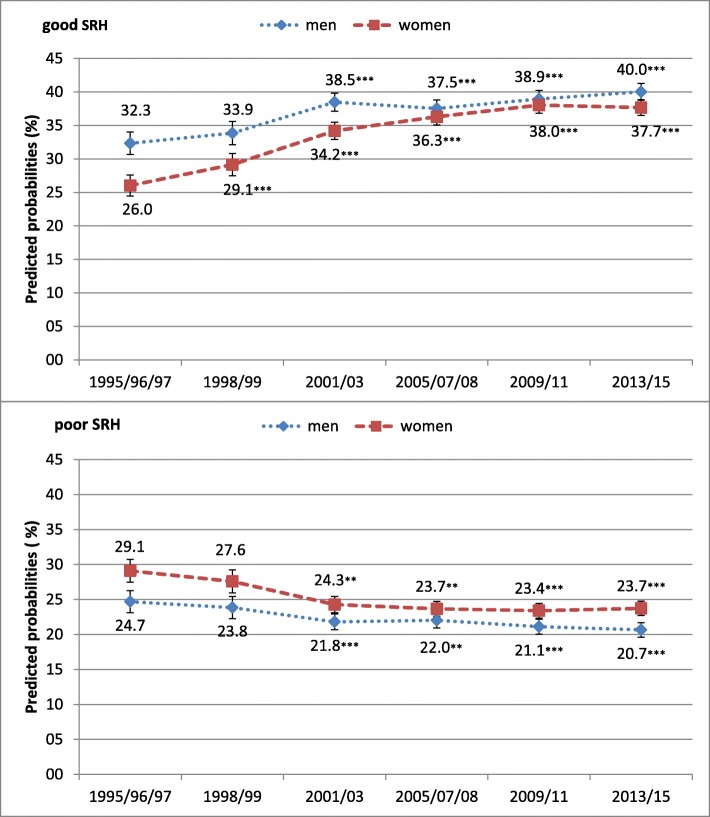
Predicted probabilities of good and poor SRH over time in men and women aged 50 to 70 years with 95% confidence intervals, adjusted for age. Significant changes in SRH compared with baseline (1995/96/97): *p < 0.05, **p < 0.01, ***p < 0.001

**Fig. 3:**
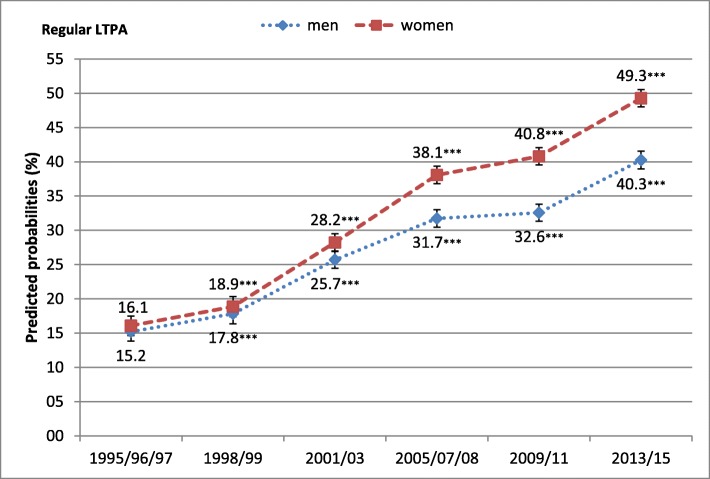
Predicted probabilities of regular LTPA in men and women over time with 95% confidence intervals, adjusted for age. Significant changes in LTPA compared with baseline (1995/96/97): *p < 0.05, **p < 0.01, ***p < 0.001

**Fig. 4:**
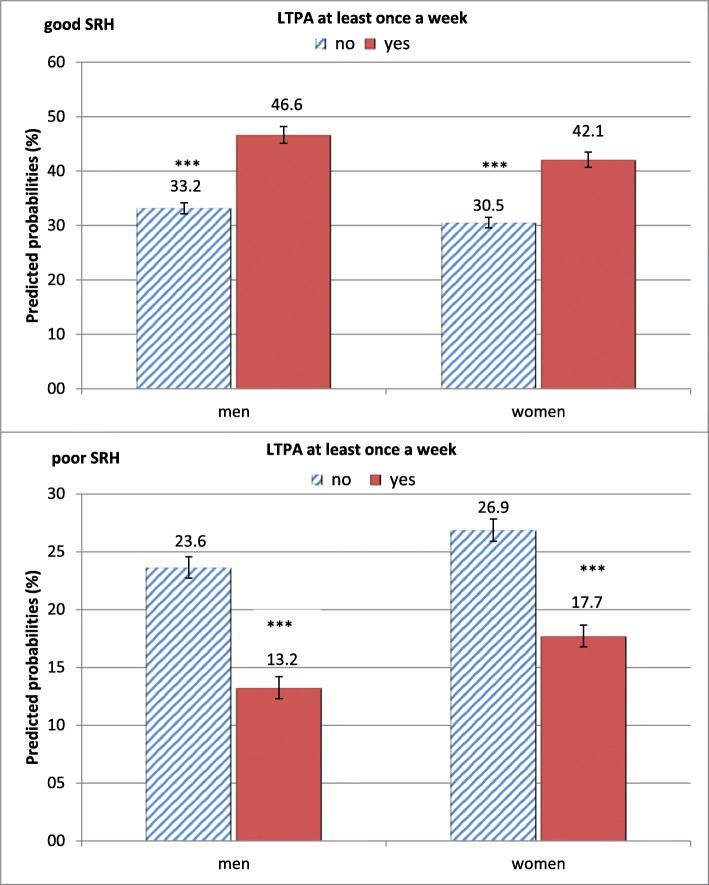
Predicted probabilities of good / poor SRH (%) according to regular LTPA in men and women with 95% confidence intervals, adjusted for age, school education and income. Significant differences between groups: *p < 0.05, **p < 0.01, ***p < 0.001

From 1995 to 2015, the predicted probability of (very) good SRH increased in men from 32.3 to 40.0% and in women from 26.0 to 37.7%. There was a simultaneous decline in the predicted probability of (very) poor SRH from 24.7 to 20.7% in men and from 29.1 to 23.7% in women (Fig. 2). In addition, the predicted probability of regular LTPA substantially increased from 15.2 to 40.3% among men and from 16.1 to 49.3% among women (Fig. 3). As Fig. 4 illustrates, the predicted probabilities of good and poor SRH differed significantly across levels of LTPA. Compared with lower levels of LTPA, being regularly physically active increased the predicted probability of good SRH from 33.2 to 46.6% in men, and from 30.5 to 42.1% in women. Conversely, the corresponding predicted probability of (very) poor SRH decreased from 23.6 to 13.2% in men and from 26.9 to 17.7% in women.

### Decomposition of time trend (good SRH)

Table 2 (upper part) reports the results of the decomposition of the total time effect on good SRH into a direct and indirect effect via temporal changes in regular LTPA. Among men (model 1), the chance of good SRH significantly increased between 1995 and 2015 (OR = 1.38). After including LTPA, the direct time effect decreased to OR = 1.14, corresponding to an indirect effect via LTPA of OR = 1.21. Expressed in average partial effects (APE), the predicted probability of good SRH increased in men by 7.3%-points over time. After controlling for temporal changes in LTPA, this increase was reduced to 2.9%-points (direct effect), leaving an increase by 4.4%-points attributable to the indirect time effect via LTPA. As the confounding ratio indicated, the total time effect was 2.5 times larger than the direct effect and 60.1% of the total time effect was due to increases in regular LTPA over time (Confounding percentage: Conf_pct). Controlling for socioeconomic status (SES) (model 2) led to a considerably decreased but still significant total time effect (OR = 1.13 / APE = 2.7%-points) which could be fully assigned to the indirect effect of regular LTPA.

**Table 2:** Decomposition of the total time effect on good / poor SRH into direct and indirect effects via leisure time physical activity (LTPA) – using KHB-method for GEE logistic regression

	Good SRH							
	Men				Women			
	Model 1		Model 2		Model 1		Model 2	
	OR	95% CI	OR	95% CI	OR	95% CI	OR	95% CI
Total time effect	1.38***	1.25–1.52	1.13*	1.04–1.29	1.77***	1.63–1.98	1.34***	1.20–1.41
Direct time effect	1.14*	1.03–1.25	1.00	0.93–1.15	1.38***	1.26–1.52	1.15**	1.03–1.28
Indirect time effect	1.21***	1.19–1.24	1.13***	1.11–1.15	1.28***	1.24–1.31	1.17***	1.14–1.19
	APE (%-points)	95% CI	APE (%-points)	95% CI	APE (%-points)	95% CI	APE (%-points)	95% CI
Total time effect	7.3	5.1–9.4	2.7	0.9–5.7	12.4	10.4–14.4	6.8	4.5–9.1
Direct time effect	2.9	0.7–5.1	0.0	−1.6-3.1	7.1	5.0–9.2	3.5	1.2–5.9
Indirect time effect	4.4	---^a^	2.7	--- ^a^	5.3	--- ^a^	3.3	--- ^a^
Conf_ratio	2.5		--- ^b^		1.7		2.1	
Conf_pct	60.1		100.0		42.8		53.4	
	Poor SRH							
	Men				Women			
	Model 1		Model 2		Model 1		Model 2	
	OR	95% CI	OR	95% CI	OR	95% CI	OR	95% CI
Total time effect	0.75***	0.67–0.84	1.04	0.91–1.18	0.72***	0.65–0.80	0.99	0.88–1.12
Direct time effect	0.96	0.86–1.08	1.21**	1.07–1.38	0.92	0.83–1.03	1.18**	1.04–1.33
Indirect time effect	0.79***	0.77–0.81	0.86***	0.84–0.88	0.78***	0.76–0.80	0.85***	0.83–0.87
	APE (%-points)	95% CI	APE (%-points)	95% CI	APE (%-points)	95% CI	APE (%-points)	95% CI
Total time effect	−4.7	−6.6-2.8	0.6	−1.4-2.6	−6.0	−8.2-0.4	−0.1	−0.22-0.20
Direct time effect	−0.7	−2.6-1.2	3.0	1.0–5.0	−1.4	−3.5-0.5	2.8	0.70–4.94
Indirect time effect	−4.0	--- ^a^	−2.4	--- ^a^	−4.6	--- ^a^	−2.9	--- ^a^
Conf_ratio	6.9		--- ^c^		4.2		--- ^c^	
Conf_pct	85.5		--- ^c^		76.3		--- ^c^	

*OR* Odds ratio, *APE* Average partial effects (change in average probability of good / poor health over time in percentage points), model 1 adjusted for age, model 2 additionally adjusted for school education, income and occupational position. Conf_ratio: Confounding ratio, gives information on the total effect size relative to the direct effect size, calculated by: total effect / direct effect. Conf_pct: Confounding percentage measures the percentage change of effect attributable to confounding net of rescaling, calculated by: indirect effect / total effect. *p < 0.05, **p < 0.01, ***p < 0.001,--- ^a^95% Confidence intervall cannot be calculated since standard errors of indirect effects are not known for APE method, −--^b^Conf_ratio cannot be calculated in case of direct effect = 0, −--^c^Conf_ratio and Conf_pct cannot be meaningfully interpreted in case of non-significant total effect

Among women, the chance of good SRH increased more strongly over time (OR = 1.77 / APE = 12.4%-points) (model 1). Of this increase, 7.1%-points were attributable to the direct effect while 5.3%-points could be assigned to the indirect effect of regular LTPA. In women, the total time effect was 1.7 times larger than the direct effect and 42.8% of the total effect could be explained by changes in regular LTPA. Controlling for SES (model 2) led to a substantially lower but still highly significant total time effect (OR = 1.34). 3.5%-points of the total remaining increase (APE = 6.8%-points), were attributable to the direct and 3.3%-points to indirect effect. This means that after controlling for age and SES, 53.4% of the total time effect was due to increases in regular LTPA over time.

### Decomposition of time trend (poor SRH)

The predicted probability of poor SRH significantly decreased over time for both, men (OR = 0.75 / APE = − 4.7%-points) and women (OR = 0.72 / APE = − 6.0%-points) (Table 2, lower part). Decomposing the total time effect resulted in a non-significant direct effect in both genders (men: OR = 0.96 / APE = − 0.7%-points; women: OR = 0.92 / APE = − 1.4%-points) while the indirect effect of regular LTPA revealed to be significant (men: OR = 0.79 / APE = − 4.0%-points; women: OR = 0.78 / APE = − 4.6%-points). Regular LTPA accounted for 85.5% of the total time effect in men and for 76.3% in women. Controlling for SES (model 2) led to a distinct decrease of the total time effect in both genders (men: OR = 1.04 / APE = 0.6%-points; women: OR = 0.99 / APE = − 0.1%-points). By disentangling the total time effect it turned out that the direct time effect pointed to a significant increase while the indirect effect indicated a significant decrease of poor SRH for both genders. These findings suggested a suppression effect of regular LTPA that concealed the total time effect. Due to suppression effect, the confounding ratio and confounding percentages could not be meaningfully interpreted.

## Discussion

In order to improve public finance sustainability, the pension age in Germany will gradually increase up to 67 years in the year 2031. We focused on individuals aged 50 to 70 years as this age group is most directly affected by changes of pension policy. Our findings highlight that the positive temporal trend in SRH among senior workers at population level can partially be attributed to increasing rates of at least weekly LTPA.

We found the three preconditions for a mediation effect of regular LTPA on trend in SRH to be met. First, in line with previous studies pointing to compression of morbidity in Germany [9–13] we found that the predicted probabilities of good SRH were increasing while those of poor SRH decreased over time in both genders. The major increase was seen in the first 8 years, while after 2001/03 this trend has been weakening for both genders, in particular for men. Secondly, in accordance with findings from studies in other countries [22–25] we found a marked increase in the predicted probability of regular LTPA over the study period for both genders. Thirdly, also in coincidence with earlier findings, regular LTPA was associated with improved SRH [16–21].

So far, most studies on the health effect of regular LTPA have focused on individual health trajectories. In this context Rabel et al. [37] found that a change to a physically more active lifestyle was positively associated with improved physical and mental health-related quality of life. However, so far the mediating effect of LTPA on health trends at population level has rarely been analyzed. After adjusting for SES, we found that the time effect on SRH significantly decreased, indicating that temporal changes in the distribution of school education, income and occupational position accounted for improvements in SRH. Changes in socio-economic conditions due to the financial crisis in 2008 might have contributed to the decline of proportions of good SRH observed in our study at that time in particular among men. Further attention should be given to the impact of socio-economical changes on temporal trends in SRH and their possible differential effect for men and women.

Adjusted for SES, we found that more than half of the remaining time effect on good SRH in women and the fully remaining smaller effect in men could be attributed to increased regular LTPA over time. With respect to poor health it turned out that the time effect on SRH disappeared after adjusting for SES, however reappeared after including regular LTPA in the model. This situation in which the magnitude of the relationship between two variables becomes larger in the presence of a third variable would indicate suppression [38]. In our case, the suppression effect of regular LTPA indicated that (after controlling for SES) a decline in SRH would have taken place when regular LTPA had not increased over time. Hence, we found regular LTPA to be mediating the temporal development of good as well as poor SRH. However, given the lack of research in this field, further studies are warranted in order to validate our findings.

According to the WHO one of the myths of aging is that it is too late to adopt a healthy lifestyle in the later years. On the contrary, it is argued that engaging in appropriate physical activity can prevent functional decline and enhance one’s quality of life even in older age [39]. In line with this assumption, this study provides first evidence that increasing LTPA over time at population level might contribute to improve SRH of elderly workers. Therefore, promoting LTPA might be a key factor to maintain work ability into old age. Since LTPA is just one of the possible influencing factors affecting health and well-being, further studies should also focus on other determinants contributing to change of SRH at population level. Within a conceptual framework for explaining socioeconomic inequalities in health, structural, psychosocial and behavioral factors were found to be important pathways [40]. Moving the field of research forward, this conceptual framework might not only be applicable to cross-sectional analyses but could also be useful for explaining health trends and the development of health inequalities over time.

Finally, some important limitations of our study should be addressed. First, as all measures used were self-reported, they are subject for potential measurement error [41]. Lacking information on the institutionalized population and persons who could not participate in the survey for health reasons may have led to an overestimation of proportions of good SRH [42]. In addition, it has to be considered that changes in the perception of health over time may contribute to changes in SRH. Due to possible social desirability bias, self-reports of LTPA may led to an under-reporting of low levels of LTPA. Furthermore, in accordance with longitudinal data demonstrating long-term health benefits of physical activity, we assumed that LTPA affected SRH [16]. However, firm conclusions cannot be drawn for the causal relationship as our analyses based on cross-sectional comparisons. Hence, it might also be possible that increasing SRH over time has led to rise in LTPA. Finally, the single item used for measuring ‘leisure time sports activities’ does not allow for a distinction between moderate and vigorous LTPA and for answering the question of whether LTPA has changed according to the proportion of subjects achieving physical activity recommendations, for instance made by the WHO [43].

## Conclusion

We found a substantial part of the health trend in SRH from 1995 to 2015 among the elderly population in Germany to be explained by increases in regular LTPA. Our results support the view that promoting regular LTPA may help to increase the number of elderly workers in the labor market. Given the importance of regular LTPA, public health policies should promote the working and environmental conditions that facilitate the integration of exercise in everyday living. Since healthy aging depends not only on behavioral but on a variety of social, cultural, economic and personal determinants, equal priority should be given on policies that focus on disease prevention and health promotion, aiming at developing both individual capabilities and healthy environments.

## Data Availability

The raw data were drawn from the German Socio-Economic Panel Study (GSOEP 21 V.31). The datasets used during the current study are available from the corresponding author on reasonable request. German data privacy laws necessitate that all users sign a data user contract with DIW Berlin.

## Abbreviations

APE: Average partial effects
GEE: Generalized Estimation Equation
GSOEP: German Socio-Economic Panel
KHB method: Karlson-Holm-Breen method
LTPA: Leisure time physical activity
OR: Odds ratio
PA: Physical activity
SRH: Self rated health
